# Family Caregiver Experiences Coordinating Care of Older Adults

**DOI:** 10.1001/jamanetworkopen.2025.44315

**Published:** 2025-11-19

**Authors:** Jennifer L. Wolff, Chanee D. Fabius, M. J. Wu, Vicki A. Freedman

**Affiliations:** 1 Department of Health Policy and Management, Johns Hopkins Bloomberg School of Public Health, Baltimore, Maryland; 2 Institute for Social Research, University of Michigan, Ann Arbor

## Abstract

**Importance:**

Older adults with disabilities rely on support from family caregivers, medical professionals, and paid caregivers. Unlike team-based interprofessional care, coordination of care work is not protocolized or well understood.

**Objective:**

To describe family caregiver experiences coordinating care with medical professionals, other family caregivers, and paid caregivers.

**Design, Setting, and Participants:**

This cross-sectional analysis of the 2023 National Health and Aging Trends Study and the linked National Study of Caregiving included data on 2811 family and unpaid caregivers to community-living adults aged 65 years or older with disabilities.

**Exposure:**

Caregiver characteristics, caregiving circumstances, negative consequences, supportive services use, and, for medical professionals, frequency of interactions and types of communication behaviors.

**Main Outcomes and Measures:**

Adapted relational coordination index (RCI; range, 1-5; greater values indicate higher relational coordination) reflecting frequency of any communication and problem-solving communication, as well as shared goals, shared knowledge, and mutual respect with medical professionals, paid caregivers, and other family caregivers.

**Results:**

Among a weighted sample of 2811 family and unpaid caregivers (1633 women [63.4%]; 1044 aged ≥65 years [41.5%]), most (2448 [87.6%]) engaged in care work that involved medical professionals (2061 [76.4%]), other family caregivers (1791 [62.8%]), or paid caregivers (822 [23.2%]). Relational coordination was reported to be weak with medical professionals (RCI, 3.10 [95% CI, 3.03-3.16]) and moderate for paid caregivers (RCI, 3.80 [95% CI, 3.71-3.89]) and other family caregivers (RCI, 4.20 [95% CI, 4.13-4.27]). Across all roles, relational coordination was higher when caregivers were related to the older adult, assisted an older adult with greater care needs, and engaged in higher-intensity caregiving. Use of supportive services was associated with higher relational coordination with medical professionals and paid caregivers but varied in magnitude by service type; the greatest differential was observed for family caregivers who did (vs did not) receive training when reflecting on medical professionals (3.63 [95% CI, 3.42-3.83] vs 3.05 [95% CI, 2.99-3.12]; *P* < .001). The RCI fluctuated from 2.33 [95% CI, 2.24-2.42] among caregivers who never interacted with medical professionals to 4.21 [95% CI, 4.11-4.31] among caregivers who interacted with medical professionals often and were always or usually asked about needed help.

**Conclusions and Relevance:**

This cross-sectional study of a national survey found that few family caregivers engage in care work alone. Further study is needed to determine if weak relational coordination with medical professionals may be remediated through training and supportive communication.

## Key Points

**Question:**

What are family caregiver experiences coordinating care of older adults?

**Findings:**

This cross-sectional study of a national survey found that few family caregivers engage in care work alone and that family caregivers rated coordination of care with medical professionals as weak and rated coordination of care with paid caregivers and other family caregivers as moderate. Coordination of care with medical professionals was rated more highly in the presence of role-related training, more frequent interactions, and supportive communication.

**Meaning:**

Further study is needed to determine if weak coordination of care between family caregivers and medical professionals may be remediated through role-related training and frequent and supportive communication.

## Teaser

This cross-sectional study uses data from the 2023 National Health and Aging Trends Study and the linked National Study of Caregiving to describe family caregiver experiences coordinating care with medical professionals, other family caregivers, and paid caregivers.

## Introduction

Community-living older adults with disabilities commonly receive help from family and unpaid caregivers (hereafter, *family caregivers*).^1^ Family caregivers provide hands-on assistance^2^ and help with health care tasks such as physician visits,^3^ medical decision-making,^4,5^ and coordinating care with medical professionals, other family caregivers, and paid caregivers.^6^ Prior work demonstrates challenges imposed on family caregivers by the fragmented service delivery environment,^3,7^ and that the frequency and quality of communication with medical professionals is variable.^5^ Because family caregivers are not systematically identified or routinely assessed in care delivery,^8,9,10^ knowledge of their experiences interacting and coordinating with other care professionals (hereafter, *coordinating care*) is limited.

A better understanding of family caregiver perspectives on coordinating care is timely and needed. Reinforced by consensus of the importance of care work to a high-value delivery system,^11^ payment policies seek to more effectively integrate and support family and paid caregivers as care team members.^12,13,14^ Older adults with disabilities often rely on more than one family caregiver, and many also rely on paid caregivers and have multiple clinicians, amplifying the need to coordinate care.^15,16^ The composition of care networks can affect care quality and family caregiver experiences, but are not well characterized.^15,17^ Moreover, interprofessional teamwork has been recognized as foundational to safe and high-quality care in later life,^18^ yet the teams—and roles—involved in care work are typically not deliberatively assembled, recognized, or well understood.

This study draws on items introduced in the 2023 National Study of Caregiving (NSOC) that afford the first national description of family caregiver experiences relating and communicating for the purposes of coordinating care. Relational coordination theory proposes that frequent, timely, accurate, problem-solving communication, reinforced by relationships that are built on shared goals, shared knowledge, and mutual respect, can best support task integration and performance outcomes.^19^ Strong evidence from diverse sectors demonstrates that a 7-item composite measure of relational coordination (the relational coordination index [RCI]) is positively associated with performance outcomes such as patient safety, organizational efficiency, and clinician burnout—and that relational coordination is modifiable.^19,20^ Thus, the RCI characterizes relational coordination involving care coordination for older adults as perceived by family caregivers; the extent to which it varies may uncover new arenas of clinical and policy intervention for older adults and their caregivers.

## Methods

### Data Sources

Data were drawn from the 2023 National Health and Aging Trends Study (NHATS) and NSOC, 2 linked national surveys that provide information for a well-defined population of older adults and their family caregivers. The NHATS and NSOC were approved by the Johns Hopkins University and University of Michigan institutional review boards, respectively. Both studies obtained written informed consent from participants. This secondary analysis used deidentified, publicly available data; thus, institutional review board review was not required in accordance with the Common Rule. This study followed the Strengthening the Reporting of Observational Studies in Epidemiology (STROBE) reporting guideline for cross-sectional studies.

The NHATS is nationally representative of individuals in the US aged 65 year or older (aged 66 years or older in 2023). In-person interviews are conducted with study participants or with proxy respondents if the participant is unable to respond. Study participants are asked whether and how they performed daily activities in the month prior to the interview. Among older adults receiving assistance, a roster lists the relationship and specific activities for each person providing help.

The NSOC is a nationally representative survey of family and unpaid caregivers to older adults who receive assistance for health and functioning reasons. The NHATS first identifies participants who live in the community and receive help with mobility, self-care, or household activities for health and functioning reasons or who live in a residential care facility with supportive services. Caregivers to these NHATS participants are eligible if they are a family member or unpaid nonrelative assisting with mobility, self-care, household activities (including shopping and helping with money), transportation, or medical tasks (such as attending medical visits and managing medications). The NSOC is conducted by internet or telephone with up to 5 eligible caregivers for each older adult. For older adults with more than 5 eligible caregivers, 5 are selected at random, with the remaining considered ineligible.

Of 8597 NHATS participants in 2023, 2637 had caregivers eligible for the NSOC, and 5456 caregivers met eligibility criteria for the NSOC. Nonresponse to the NSOC can arise from the NHATS participant (who may refuse to provide contact information for caregivers) or their family caregivers (who may refuse to participate). NHATS participants did not provide contact information for 420 eligible family caregivers and 2130 of the remaining 5036 eligible family caregivers could not be located or refused to respond, yielding a 92.3% first-stage response rate and a 57.7% second-stage response rate. We focused on the sample of 2906 family caregiver respondents who assisted 1904 living NHATS participants. We then excluded 61 NSOC respondents who were assisting older adults living in a nursing facility and 34 who did not help in the last month, leaving a final analytic sample of 2811 family caregivers to 1857 community-living older adults.

### Measures

The RCI is a 7-item composite measure of the relational dynamics among individuals occupying specified roles for a given work process.^19,21^ The NSOC tailored the RCI items with permission from Relational Coordination Analytics, Inc, by asking respondents about 3 roles that are common in care work involving older adults: medical professionals (referred to in the NSOC as *medical providers*), paid caregivers, and (other) family caregivers (Table 1).^5,6,22^ For each role identified as involved in the older adult’s care, the NSOC respondent was asked about 2 communication domains (frequency and problem solving; timeliness and accuracy from the original instrument were excluded) and 3 relationship domains (shared goals, shared knowledge, and mutual respect). The original instrument uses a 5-point Likert scale and 6-month reference period. To minimize mode effects^23^ and align with the NSOC instrument, a 4-item response scale and a 1-month recall period were used. For problem-solving communication, a fifth category was offered to indicate no care-related problems.

**Table 1.** Relational Coordination Items Fielded in 2023 National Study of Caregiving^a^

Construct	Measure	Response
Role enumeration	In the last month that you helped [SP] did any of the following types of people provide care to [SP]? (1) Medical provider,^b^ (2) Paid caregiver, (3) Not counting you, another family member or friend?	Yes/no
Frequency of communication	In the last month, how often did you communicate with [SP’s] (1) Medical providers? (2) Paid caregivers? (3) Other caregivers who were family or friends?	Often, sometimes, rarely, never
Problem-solving communication	In the last month, how often did [SP’s] (1) Medical providers, (2) Paid caregivers, (3) Other caregivers who were family or friends work with you to solve problems related to [SP’s] care?	Often, sometimes, rarely, never, did not have problems
Shared knowledge	In the last month, how much did [SP’s] (1) Medical providers, (2) Paid caregivers, (3) Other caregivers who were family or friends know about what you do for [SP]?	A lot, somewhat, a little, not at all
Mutual respect	In the last month, how much did [SP’s] (1) Medical providers, (2) Paid caregivers, (3) Other caregivers who were family or friends respect your role in [SP’s] care?	A lot, somewhat, a little, not at all
Shared goals	In the last month, how much did [SP’s] (1) Medical providers, (2) Paid caregivers, (3) Other caregivers who were family or friends know about what you do/did for [SP]?	A lot, somewhat, a little, not at all

Abbreviations: NHATS, National Health and Aging Trends Study; NSOC, National Study of Caregiving; RCI, relational coordination index; SP, study participant.

^a^ Family caregivers are asked the 5 RCI items for each of the 3 specified groups (medical providers, paid caregivers, other family member or friend caregivers) identified (in the role enumeration question) as providing care to the NHATS SP. The adapted RCI represents an equally weighted index of 5 items—see NSOC technical paper^22^ for details.

^b^ The description in the survey for *medical provider* states: “This may be a doctor, nurse, physician’s assistant, physical therapist, hospice provider, or palliative care specialist.”

We followed established procedures^21^ to create a summary measure of relational coordination for each role, reflecting the NSOC respondent’s view of the care network.^22^ We first constructed a separate analytic sample for each role, inclusive of respondents reporting involvement of medical professionals, other family caregivers, or paid caregivers in the NHATS participant’s care. We excluded a small number of respondents (medical professionals, 33 of 2061 [1.6%]; other family caregivers, 22 of 1791 [1.2%]; paid caregivers, 11 of 822 [1.3%]) completing fewer than 3 of the 5 RCI items within a given role. Respondents with 1 or 2 missing RCI items were assigned the modal value of each missing item.

Relational coordination items loaded on a single factor, demonstrating strong internal consistency by role for caregivers who reported problem solving and for those who did not (Cronbach α ≥ 0.85 for 5 items and ≥0.81 for 4 items). We then constructed a composite measure from standardized item means for each role. We recalibrated response categories for the composite measure (from a standardized mean to a scale from 1 to 5) to provide comparability in evaluating cut points classifying the strength of ties within and between roles (within-role ties tend to be stronger). Established cut points for the interpretation of between-role scoring (in this analysis, with medical professionals and paid caregivers) of less than 3.5 indicate weak ties, 3.5 to 3.99 indicate moderate ties, and 4.0 or more indicate strong ties. Established cut points for the interpretation of within-role scoring (in this analysis, with other family caregivers) are less than 4.1 (weak ties), 4.1 to 4.59 (moderate ties), and more than 4.6 (strong ties).^21^

Key measures include sociodemographic characteristics of family caregivers, older adults’ care needs, caregiving circumstances, caregiving-related negative consequences, and use of supportive services. Caregiver characteristics included age, sex, self-reported race and ethnicity (Hispanic, non-Hispanic Black, non-Hispanic White, and other race or ethnicity [Alaska Native, American Indian, Asian, Native Hawaiian, Pacific Islander; these categories were combined due to small samples and to adhere to reporting requirements]), educational attainment, self-rated health, and relationship to older adult. We included 2 items relating to older adults’ care needs. Dementia refers to probable dementia, from self-reported physician diagnosis of Alzheimer disease or dementia, the AD8 (Ascertain Dementia 8) Dementia Screening Interview (administered to proxy respondents),^24^ and cognitive tests to evaluate memory, orientation, and executive function.^25^ We also included a measure reflecting the number of self-care and mobility activities with which the older adults received help (1-2, 3-4, and 5-7).

Caregiving circumstances included travel time between caregiver’s and older adult’s residences, duration of caregiving (eg, in years), hours of care in the previous week, and types of assistance provided—all of which are associated with the frequency and nature of communicating and relating when coordinating care. Negative consequences, including physical, financial, and emotional difficulties due to caregiving, were based on reported level of difficulty, contrasting little or none (0 or 1 on 5-point Likert scale) with some or substantial (≥2 on 5-point Likert scale). Participation restrictions refer to activities that were limited in the prior month due to caregiving and stated as very or somewhat important to the caregiver. Work productivity loss reflects the presence of absenteeism (missed hours of work due to caregiving in relation to typical hours worked) or presenteeism (impact of caregiving on productivity while at work) among caregivers who reported working for pay.^26^ Caregivers’ use of supportive services was examined using a 1-year reference period.

Finally, we analyzed NSOC items specific to older adults’ medical professionals (referred to in the NSOC as *medical providers*), as previously described,^5^ to enable granular analyses on perspectives of care coordination with medical professionals. Family caregivers were categorized as never, sometimes or rarely, or often interacting with medical professionals. Communication behaviors were measured using 3 questions that were asked of caregivers who interacted with older adults’ medical professionals. These caregivers were told to think about the medical professional they communicated with most often and were then asked: “In the last year, how often did that [professional]” … “listen to what you had to say,” “ask if you understood [older adult’s] health treatments,” and “ask if you needed help managing [older adult’s] health treatments?” For these 3 dimensions, we contrast responses of always or usually with sometimes or never.

### Statistical Analysis

We examined family caregiver perspectives on coordinating older adults’ care with medical professionals, paid caregivers, and other family caregivers. We first described characteristics of family caregivers of older adults receiving care from medical professionals, paid caregivers, and other family caregivers. We then described relational coordination reported by family caregivers when reflecting on each role. Here we descriptively reported mean RCI values for each role by family caregiver and older adult characteristics. We then examined mean RCI values across dimensions of caregiving circumstances (eg, tasks, intensity), negative consequences (caregiving-related emotional, physical, and financial difficulty; participation restrictions; work productivity loss), and use of supportive services. Finally, we reported mean RCI values by family caregivers’ frequency and experiences with older adults’ medical professionals.

When reporting on dimensions of caregiving circumstances and negative consequences, we reported adjusted mean RCIs that account for older adults’ dementia status and count of activity limitations—recognizing the importance of care demands to caregiving experiences. Item nonresponse was generally low (<1.0% for covariates except caregiver age [2.8%], travel time [2.2%], and education [2.0%]). Missing values were recoded to modal values. We use *P* value cut points of <.01 to denote significance in results. Analyses were conducted in R, version 4.4.1 (R Project for Statistical Computing),^27^ using survey sampling weights and procedures that account for the NSOC’s complex sample design; population estimates were poststratified to align with those from the US Census.^28^

## Results

Among the weighted sample of 2811 family and unpaid caregivers (1633 women [63.4%] and 815 men [36.6%]; 1044 aged ≥65 years [41.5%]; 394 Hispanic individuals [9.9%], 619 non-Hispanic Black individuals [14.5%], 1298 non-Hispanic White individuals [69.3%], and 137 individuals of other race and ethnicity [6.3%]) assisting older adults in 2023, most (2448 [87.6%]) assisted an older adult who was receiving help from medical professionals, other family caregivers, or paid caregivers (Figure). Family caregivers reported medical professionals (2061 [76.4%]) and other family caregivers (1791 [62.8%]) to be most often present; paid caregivers (822 [23.2%]) were less commonly involved. Family caregivers who were engaged in care coordination with paid caregivers were more likely to be aged 55 to 64 years and daughters or sons assisting an older adult with greater care needs involving dementia, or receiving help with greater numbers of self-care or mobility activities (eTable in Supplement 1). Spousal caregivers were most likely to be engaged in care coordination with medical professionals and nonrelatives were least likely to be engaged in care coordination with paid caregivers—but few other notable differences were observed.

**Figure.**
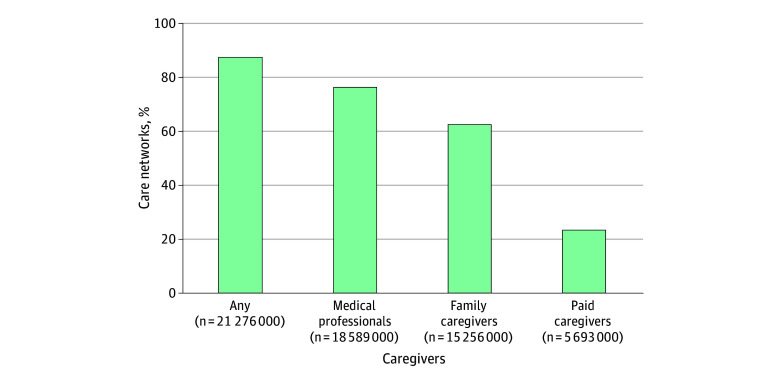
Older Adults’ Care Networks (Family Caregiver and Other Roles)

Family caregiver perspectives of relational coordination were rated as weak when reflecting on coordinating care with medical professionals (RCI, 3.10 [95% CI, 3.03-3.16]) and moderate for paid caregivers (RCI, 3.80 [95% CI, 3.71-3.89]) and other family caregivers (RCI, 4.20 [95% CI, 4.13-4.27] (Table 2). Relational coordination was rated as lower among family caregivers who were not related to the older adult and when assisting older adults with fewer care needs—both cognitive (by dementia status) and physical function (by numbers of self-care or mobility limitations).

**Table 2.** Family Caregiver–Reported Relational Coordination, Stratified by Role

Caregiver characteristic	Medical professionals		Caregivers			
			Other family		Paid	
	RCI (95% CI)	*P* value	RCI (95% CI)	*P* value	RCI (95% CI)	*P* value
Total	3.10 (3.03-3.16)	NA	4.20 (4.13-4.27)	NA	3.80 (3.71-3.89)	NA
Age, y						
<55	3.09 (2.98-3.19)	.04	4.33 (4.22-4.44)	.01	3.86 (3.70-4.02)	.63
55-64	3.22 (3.06-3.38)		4.19 (4.06-4.32)		3.79 (3.62-3.96)	
65-74	3.10 (2.98-3.22)		4.06 (3.91-4.21)		3.72 (3.56-3.88)	
≥75	2.93 (2.80-3.07)		4.07 (3.88-4.26)		3.79 (3.56-4.02)	
Sex						
Male	3.05 (2.94-3.17)	.30	4.16 (4.05-4.27)	.33	3.70 (3.57-3.83)	.06
Female	3.12 (3.05-3.19)		4.22 (4.13-4.31)		3.86 (3.74-3.98)	
Race and ethnicity						
Hispanic	3.27 (3.01-3.54)	.01	4.13 (3.98-4.28)	.002	3.99 (3.74-4.24)	.03
Non-Hispanic Black	3.28 (3.16-3.39)		4.35 (4.29-4.41)		3.91 (3.73-4.09)	
Non-Hispanic White	3.05 (2.96-3.14)		4.17 (4.07-4.27)		3.71 (3.58-3.84)	
Other	2.92 (2.67-3.17)		4.22 (4.01-4.43)		4.09 (3.83-4.35)	
Educational attainment						
High school or less	3.14 (3.02-3.25)	.41	4.15 (4.04-4.26)	.01	3.90 (3.73-4.07)	.17
Some college	2.99 (2.81-3.18)		4.06 (3.90-4.22)		3.58 (3.34-3.82)	
College or beyond	3.13 (3.05-3.21)		4.30 (4.23-4.37)		3.83 (3.71-3.95)	
Perceived health status						
Excellent or very good	3.06 (2.97-3.16)	.14	4.23 (4.13-4.33)	.28	3.86 (3.71-4.01)	.05
Good	3.05 (2.96-3.15)		4.13 (4.01-4.25)		3.62 (3.45-3.79)	
Fair or poor	3.25 (3.07-3.44)		4.24 (4.14-4.34)		3.97 (3.76-4.18)	
Relationship to older adult						
Spouse	3.28 (3.16-3.39)	<.001	4.24 (4.13-4.35)	.001	4.03 (3.75-4.31)	≤.001
Daughter or son	3.24 (3.14-3.33)		4.28 (4.20-4.36)		3.84 (3.72-3.96)	
Other relative	2.88 (2.69-3.07)		4.27 (4.11-4.43)		3.80 (3.54-4.06)	
Nonrelative	2.45 (2.22-2.68)		3.69 (3.44-3.94)		2.99 (2.72-3.26)	
Older adult dementia status						
No dementia	3.00 (2.93-3.08)	≤.001	4.16 (4.07-4.25)	.03	3.67 (3.55-3.79)	.002
Dementia	3.37 (3.26-3.49)		4.30 (4.19-4.41)		3.99 (3.85-4.13)	
Older adult helped with^a^						
0 Self-care or mobility activities	2.90 (2.78-3.02)	<.001	4.08 (3.95-4.21)	.002	3.46 (3.16-3.76)	<.001
1-2 Self-care or mobility activities	3.03 (2.92-3.15)		4.15 (4.05-4.25)		3.58 (3.46-3.70)	
3-4 Self-care or mobility activities	3.21 (3.04-3.39)		4.32 (4.19-4.45)		3.70 (3.47-3.93)	
5-7 Self-care or mobility activities	3.45 (3.29-3.62)		4.36 (4.25-4.47)		4.19 (4.05-4.33)	

Abbreviations: NA, not applicable; RCI, relational coordination index.

^a^ Numbers of self care or mobility activities for which older adult receives help encompasses: bathing, eating, dressing, toileting, transferring, indoor mobility, and outdoor mobility.

Family caregiver hours, proximity, and types of assistance were highly associated with relational coordination (Table 3). For example, relational coordination was rated more highly for family caregivers helping more than 20 hours per week (vs ≤20 hours): 3.47 (95% CI, 3.35-3.59) vs 2.97 (95% CI, 2.90-3.05) for medical professionals, 4.46 (95% CI, 4.37-4.54) vs 4.12 (95% CI, 4.03-4.21) for family caregivers, and 4.16 (95% CI, 4.00-4.33) vs 3.66 (95% CI, 3.54-3.78) for paid caregivers. No difference was observed by caregiving duration.

**Table 3.** Family Caregiver–Reported Relational Coordination by Circumstances, Stratified by Role

Caregiving circumstance	Medical professionals		Caregiver			
			Family		Paid	
	RCI (95% CI)	*P* value^a^	RCI (95% CI)	*P* value^a^	RCI (95% CI)	*P* value^a^
Hours of caregiving/wk						
>20	3.47 (3.35-3.59)	<.001	4.46 (4.37-4.54)	<.001	4.16 (4.00-4.33)	<.001
≤20	2.97 (2.90-3.05)		4.12 (4.03-4.21)		3.66 (3.54-3.78)	
Travel time to older adults’ residence, min						
Co-reside	3.26 (3.17-3.35)	<.001	4.30 (4.21-4.38)	.01	4.01 (3.87-4.16)	.01
≤10	2.87 (2.75-2.99)		4.06 (3.93-4.20)		3.63 (3.46-3.80)	
11-30	2.96 (2.82-3.09)		4.20 (4.03-4.37)		3.69 (3.50-3.89)	
≥31	3.27 (3.13-3.41)		4.23 (4.15-4.31)		3.78 (3.55-4.01)	
Duration of caregiving, y						
<1	2.89 (2.56-3.22)	.12	4.05 (3.73-4.37)	.37	3.63 (3.31-3.96)	.31
1-3	3.06 (2.95-3.16)		4.18 (4.09-4.27)		3.74 (3.59-3.89)	
>3	3.16 (3.08-3.24)		4.23 (4.14-4.33)		3.85 (3.76-3.95)	
Types of activities						
Personal care or mobility^b^						
Yes	3.33 (3.25-3.41)	<.001	4.30 (4.21-4.39)	.005	3.99 (3.90-4.08)	<.001
No	2.80 (2.69-2.90)		4.07 (3.95-4.19)		3.39 (3.18-3.59)	
Health system logistics^c^						
Yes	3.47 (3.41-3.54)	<.001	4.36 (4.32-4.41)	<.001	4.00 (3.90-4.10)	<.001
No	2.48 (2.38-2.58)		3.99 (3.86-4.12)		3.31 (3.15-3.46)	
Health management tasks^d^						
Yes	3.31 (3.23-3.39)	<.001	4.27 (4.18-4.35)	.06	3.98 (3.87-4.09)	.001
No	2.83 (2.72-2.93)		4.12 (4.00-4.24)		3.49 (3.28-3.70)	
Health care tasks^e^						
Yes	3.43 (3.34-3.52)	<.001	4.35 (4.29-4.42)	<.001	3.99 (3.86-4.11)	<.001
No	2.72 (2.63-2.81)		4.04 (3.93-4.16)		3.51 (3.37-3.66)	

Abbreviation: RCI, relational coordination index.

^a^ RCI and *P* values are adjusted for older adults’ dementia status and function.

^b^ Personal care or mobility includes eating, showering, bathing, dressing, grooming, using the toilet, getting in and out of bed, getting around inside, and leaving the home to go outside.

^c^ Health system logistics include making appointments, ordering medications, and handling insurance.

^d^ Health management includes adhering to special diet, foot care, skin care, exercises, and dental care.

^e^ Health care tasks include keeping track of medications, giving shots or injections, and managing medical tasks such as ostomy care or intravenous catheter care.

Associations between relational coordination and caregiving-related negative consequences were variable (Table 4). Associations were consistently evident for emotional difficulty and participation restrictions, not evident for physical difficulty, and evident for caregiving-related financial difficulty for medical professionals only. Relational coordination did not differ by family caregivers’ employment status but was higher among working caregivers who incurred caregiving-related work productivity loss. Use of supportive services was positively associated with relational coordination involving medical professionals and paid caregivers but the magnitude varied by type of service. This difference was most apparent among family caregivers who did (vs did not) receive role-related training when reflecting on medical professionals (RCI, 3.63 [95% CI, 3.42-3.83] vs 3.05 [95% CI, 2.99-3.12]; *P* < .001).

**Table 4.** Family Caregiver–Reported Relational Coordination, Negative Consequences, and Supportive Services Use, Stratified by Role

Negative consequence	Medical professionals		Caregiver			
			Family		Paid	
	RCI (95% CI)	*P* value^a^	RCI (95% CI)	*P* value^a^	RCI (95% CI)	*P* value^a^
Emotional difficulty^b^						
Some or substantial	3.29 (3.17-3.41)	.001	4.28 (4.18-4.38)	.01	3.90 (3.78-4.02)	.05
Little to none	2.99 (2.91-3.08)		4.15 (4.07-4.23)		3.71 (3.58-3.84)	
Physical difficulty^b^						
Some or substantial	3.24 (3.05-3.42)	.11	4.23 (4.12-4.35)	.48	3.92 (3.75-4.10)	.13
Little to none	3.06 (2.99-3.14)		4.19 (4.11-4.27)		3.76 (3.65-3.86)	
Financial difficulty^b^						
Some or substantial	3.36 (3.21-3.52)	.002	4.31 (4.19-4.42)	.06	3.95 (3.72-4.18)	.18
Little to none	3.05 (2.98-3.13)		4.18 (4.10-4.26)		3.76 (3.66-3.87)	
Participation restrictions in valued activities^c^						
Yes	3.46 (3.36-3.57)	.009	4.31 (4.18-4.44)	.06	4.02 (3.88-4.17)	.002
No	3.01 (2.93-3.08)		4.17 (4.09-4.25)		3.70 (3.59-3.82)	
Worked for pay last week						
Yes	3.04 (2.95-3.13)	.17	4.24 (4.13-4.34)	.27	3.82 (3.69-3.95)	.69
No	3.14 (3.04-3.24)		4.16 (4.07-4.25)		3.77 (3.62-3.93)	
Work productivity loss, for those working^d^						
Yes	3.44 (3.27-3.62)	<.001	4.49 (4.42-4.56)	<.001	4.05 (3.87-4.22)	.01
No	3.05 (2.98-3.11)		4.15 (4.08-4.23)		3.75 (3.64-3.85)	
Supportive services use						
Respite care						
Yes	3.40 (3.22-3.59)	.002	4.10 (3.88-4.33)	.39	4.15 (4.04-4.25)	<.001
No	3.06 (2.99-3.13)		4.21 (4.13-4.29)		3.68 (3.57-3.79)	
Training regarding how to assist older adult						
Yes	3.63 (3.42-3.83)	<.001	4.32 (4.17-4.47)	.17	4.22 (4.06-4.38)	<.001
No	3.05 (2.99-3.12)		4.19 (4.11-4.27)		3.73 (3.63-3.83)	
Support group participation						
Yes	3.46 (3.06-3.87)	.08	4.04 (3.78-4.30)	.28	4.26 (4.00-4.52)	.001
No	3.09 (3.02-3.15)		4.20 (4.13-4.28)		3.78 (3.68-3.87)	
Use of ≥1 supportive services						
Yes	3.45 (3.31-3.60)	<.001	4.19 (4.04-4.33)	.90	4.15 (4.07-4.22)	<.001
No	3.03 (2.95-3.10)		4.20 (4.11-4.29)		3.62 (3.50-3.74)	

Abbreviation: RCI, relational coordination index.

^a^ RCI and *P* values are adjusted for older adult dementia status and function.

^b^ Little to none refers to a score of 0 or 1 on a 5-point Likert scale; some or substantial refers to ratings of 2 or more on a 5-point Likert scale.

^c^ Refers to reduced participation in the last month due to caregiving for activities identified as being somewhat or very important to the caregiver.

^d^ Work productivity loss refers to missed time from work or reduced productivity while at work due to caregiving.

Perceptions of relational coordination fluctuated by frequency of family caregiver interactions with older adults’ medical professionals: from a low of 2.33 (95% CI, 2.24-2.42) among those who never interacted with medical professionals to 3.96 (95% CI, 3.87-4.05) among those interacting with medical professionals often (eFigure in Supplement 1). Among family caregivers who did interact with medical professionals, relational coordination was characterized as weak (<3.5) for those who did so sometimes or rarely and were sometimes or never listened to (RCI, 2.75 [95% CI, 2.53-2.97]), asked about understanding (RCI, 2.88 [95% CI, 2.75-3.01]), or asked about needed help (RCI, 3.18 [95% CI, 3.07-3.29]). Relational coordination was characterized as strong (≥4.0) among family caregivers who interacted often with medical professionals and reported always or usually being listened to (RCI, 4.04 [95% CI, 3.97-4.11]), asked about understanding (4.09 [95% CI, 4.02-4.16), and asked about needed help (4.21 [95% CI, 4.11-4.31]).

## Discussion

To our knowledge, this is the first national study of family caregiver experiences coordinating care of older adults with disabilities. We found few family caregivers engaged in caregiving without involvement of medical professionals, other family caregivers, or paid caregivers. Family caregivers identified medical professionals as most commonly present in older adults’ care, yet relational coordination was characterized as weak relative to paid caregivers and other family caregivers. A dominant finding was that family caregivers rated relational coordination more highly in the context of high-intensity caregiving across dimensions of older adults’ care needs, caregiving intensity, and role-related consequences. Study results also indicate challenges among family caregivers who are less central to care processes by virtue of not being related to the older adult or not interacting with medical professionals, both of which may translate into less visibility to information and shared norms.

Relational coordination theory emphasizes role-based relationships as distinct from personal connections. The focus on roles rather than individuals is a key aspect of our study, which reinforces challenges of the current care delivery paradigm in which the family caregiver role is poorly specified. The challenges of achieving shared goals, shared knowledge, and mutual respect in work processes involving actors (family caregivers, relative to medical professionals) who lack common training, socialization, and expertise can be especially problematic within the disjointed care delivery system involving care teams that have not been formed with intentionality. In this context, our finding that relational coordination with medical professionals was strong in the context of more frequent interactions and more supportive communication behaviors suggests the feasibility of nurturing productive partnerships that extend outside the patient–medical professional relationship to involved family caregivers.

The availability and adequacy of support provided by family caregivers has important consequences for the quality of life of and service use by older individuals in the US.^11^ Advances in medicine have led to greater duration, complexity, and technical skill of care required by older adults and delivered by family caregivers, yet medical professionals do not typically assess family caregiver knowledge, understanding, or proficiency in tasks they perform.^8,9,10^ Efforts to support family caregivers can pose challenges for medical professionals when balanced with competing responsibilities and constrained time.

Payment policy and work system redesign hold potential for strengthening the partnership between medical professionals and older adults’ family caregivers. New Medicare payment models and billing codes afford reimbursement to medical professionals for the identification, screening, and education of family caregivers to older adults with chronic and disabling conditions.^12,13^ Digital health technologies can not only support differentiated identity credentials such that family caregivers may transparently access the patient’s electronic medical record and navigate health system demands but may be used to field screening assessments and deliver tailored education and referrals resources.^29,30,31,32^

### Strengths and Limitations

Several study strengths and limitations merit comment. This is the first national study of family caregiver perceptions of relational coordination in the care of older adults. We could not comment on medical professional, paid caregiver, or older adult perspectives—all of which are relevant. Nevertheless, family caregivers are well-positioned informants given their frequent interactions with medical professionals and paid caregivers,^5,6,33^ and direct care worker scope of practice constraints that inhibit active participation in medical care.^34^ The NSOC’s adapted RCI asks about family caregiver perceptions of interactions with “medical professionals” broadly, and we therefore cannot comment on specialty, gender, years in practice, duration of relationship, or communication style—all of which are known to affect interpersonal rapport and trust.^35^ Although the RCI is grounded in relational coordination theory and the NSOC’s adapted version displayed strong internal consistency of items, the number and wording of the NSOC items and response categories differ from the original instrument.

## Conclusions

This cross-sectional study of a national survey found that few family caregivers engage in care work alone. Care coordination is an important element of safe, efficient, high-quality care.^18,36^ Our findings that relational coordination with medical professionals, which was weak overall, but higher among family caregivers who received role-related training, and strong when frequent interactions and supportive communication occurred, is encouraging and suggests the merit of further research to evaluate the potential of systems-level initiatives that formalize and support the caregiver role.

## Notes

Supplement 1. **eTable.** Characteristics of Family Caregivers Who Reported Interacting With Medical Providers, Other Family Caregivers, or Paid Caregivers **eFigure.** Family Caregiver-Reported Relational Coordination With Medical Providers

Supplement 2.
Data Sharing Statement
