# Supplementary material for: Family Caregiver Experiences Coordinating Care of Older Adults
Source: JAMA Netw Open. 2025 Nov 19;8(11):e2544315. doi: 10.1001/jamanetworkopen.2025.44315 (PMC12631485; doi:10.1001/jamanetworkopen.2025.44315)
Supplement: Supplement 1. — eTable. Characteristics of Family Caregivers Who Reported Interacting With Medical Providers, Other Family Caregivers, or Paid Caregivers eFigure. Family Caregiver-Reported Relational Coordination With Medical Providers [file jamanetwopen-e2544315-s001.pdf]

Supplementary Online Content

Wolff JL, Fabius CD, Wu MJ, Freedman VA. Family caregiver experiences coordinating care of older adults. *JAMA Netw Open.* 2025;8(11):e2544315. doi:10.1001/jamanetworkopen.2025.44315

eTable. Characteristics of Family Caregivers Who Reported Interacting With Medical Providers, Other Family Caregivers, or Paid Caregivers
eFigure. Family Caregiver-Reported Relational Coordination With Medical Providers

This supplementary material has been provided by the authors to give readers additional information about their work.

© 2025 Wolff JL et al. *JAMA Network Open.*

eTable. Characteristics of Family Caregivers Who Reported Interacting With Medical Providers, Other Family Caregivers, or Paid Caregivers

Caregiver characteristics	Any	Medical Providers	Other Family	Paid Caregivers	P-Value
Unweighted sample	2448	2061	1791	822	
Age, years (%)					
Less than 55	747 (34.9%)	35.1%	38.1%	29.3%	<0.001
55-64	657 (23.6%)	23.8%	26.5%	33.6%	
65-74	615 (24.9%)	24.6%	21.2%	23.1%	
75 and older	429 (16.6%)	16.5%	14.2%	13.9%	
Sex					
Male	815 (36.6%)	36.4%	35.7%	40.2%	0.097
Female	1633 (63.4%)	63.6%	64.3%	59.8%	
Race-ethnicity					
Non-Hispanic White	1298 (69.3%)	70.9%	68.9%	65.3%	0.223
Non-Hispanic Black	619 (14.5%)	14.2%	14.9%	18.2%	
Hispanic	394 (9.9%)	9.4%	10.5%	10.6%	
Other	137 (6.3%)	5.5%	5.7%	5.8%	
Educational attainment [b]					
High school or less	702 (28.2%)	26.4%	27.0%	24.5%	0.022
Some college	597 (24.7%)	24.6%	25.9%	20.9%	
College or beyond	1149 (47.1%)	49.0%	47.1%	54.6%	
Perceived health status					
Excellent or very good	1117 (45.4%)	45.0%	43.5%	45.2%	0.722
Good	840 (34.8%)	35.2%	36.0%	36.2%	
Fair or poor	491 (19.8%)	19.8%	20.5%	18.6%	
Relationship to older adult:					
Spouse	499 (22.0%)	23.6%	13.5%	14.2%	<0.001
Daughter or son	1368 (48.5%)	48.4%	54.6%	58.8%	
Other relative	386 (17.5%)	16.4%	19.4%	19.2%	
Nonrelative	195 (12.1%)	11.6%	12.4%	7.8%	
Older adult has dementia					
No dementia	1698 (74.0%)	74.7%	72.5%	59.5%	<0.001
Dementia	750 (26.0%)	25.3%	27.5%	40.5%	
Older adult helped with: [a]					
0 Self-care/mobility activities	606 (25.1%)	24.9%	25.5%	13.6%	<0.001
1-2 Self-care/mobility activities	989 (43.2%)	43.4%	41.1%	34.3%	
3-4 Self-care/mobility	391 (15.0%)	15.2%	15.3%	17.3%	
5-7 Self-care/mobility	462 (16.7%)	16.6%	18.0%	34.8%	

Abbreviations: std, standard error of mean.

[a] Bathing, eating, dressing, toileting, transferring, indoor mobility, outdoor mobility.

© 2025 Wolff JL et al. *JAMA Network Open.*

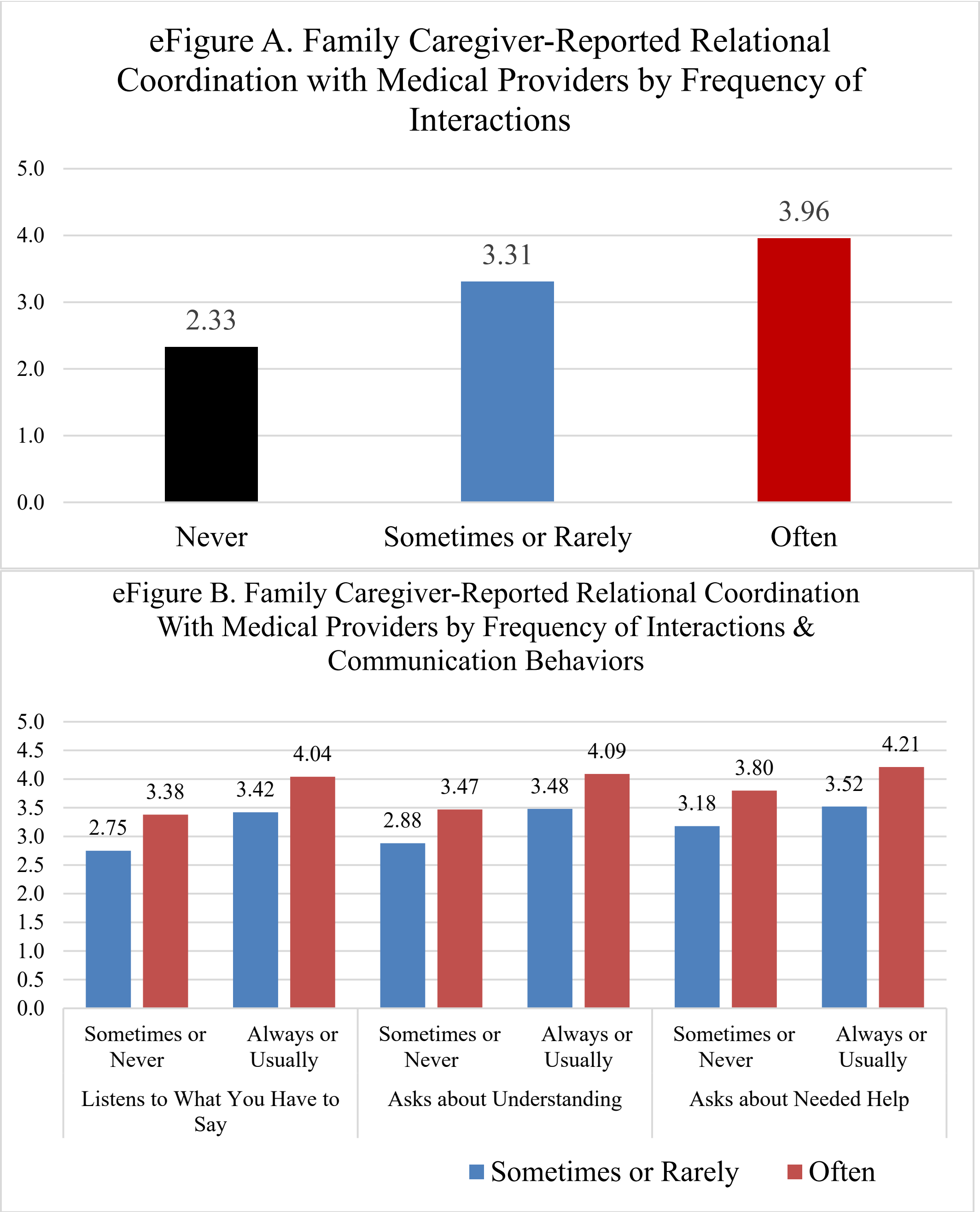

eFigure. Family Caregiver-Reported Relational Coordination With Medical Providers

© 2025 Wolff JL et al. *JAMA Network Open.*
