# Supplementary material for: Family Caregiver Experiences Coordinating Care of Older Adults
Source: JAMA Netw Open. 2025 Nov 19;8(11):e2544315. doi: 10.1001/jamanetworkopen.2025.44315 (PMC12631485; doi:10.1001/jamanetworkopen.2025.44315)
Supplement: Supplement 2. — Data Sharing Statement [file jamanetwopen-e2544315-s002.pdf]

Data Sharing Statement

Wolff. Family Caregiver Experiences Coordinating Care of Older Adults. *JAMA Netw Open*. Published November 19, 2025. doi:10.1001/jamanetworkopen.2025.44315

Data
Data available: No

Additional Information
Explanation for why data not available: Data are publicly available and we have produced a technical report describing our approach.
